# Understanding the Pathophysiology of Congenital Vestibular Disorders: Current Challenges and Future Directions

**DOI:** 10.3389/fneur.2021.708395

**Published:** 2021-09-10

**Authors:** Kenna D. Peusner, Nina M. Bell, June C. Hirsch, Mathieu Beraneck, Anastas Popratiloff

**Affiliations:** ^1^Department of Neurology, The George Washington University School of Medicine and Health Sciences, Washington, DC, United States; ^2^Université de Paris, Integrative Neuroscience and Cognition Center, CNRS UMR 8002, Paris, France; ^3^The George Washington University Nanofabrication and Imaging Center, Washington, DC, United States

**Keywords:** inner ear imaging, developmental balance disorders, vestibular system development, inner ear abnormalities, animal models

## Abstract

In congenital vestibular disorders (CVDs), children develop an abnormal inner ear before birth and face postnatal challenges to maintain posture, balance, walking, eye-hand coordination, eye tracking, or reading. Only limited information on inner ear pathology is acquired from clinical imaging of the temporal bone or studying histological slides of the temporal bone. A more comprehensive and precise assessment and determination of the underlying mechanisms necessitate analyses of the disorders at the *cellular* level, which can be achieved using animal models. Two main criteria for a suitable animal model are first, a pathology that mirrors the human disorder, and second, a reproducible experimental outcome leading to statistical power. With over 40 genes that affect inner ear development, the phenotypic abnormalities resulting from congenital vestibular disorders (CVDs) are highly variable. Nonetheless, there is a large subset of CVDs that form a common phenotype of a sac-like inner ear with the semicircular canals missing or dysplastic, and discrete abnormalities in the vestibular sensory organs. We have focused the review on this subset, but to advance research on CVDs we have added other CVDs not forming a sac-like inner ear. We have included examples of animal models used to study these CVDs. Presently, little is known about the central pathology resulting from CVDs at the cellular level in the central vestibular neural network, except for preliminary studies on a chick model that show significant loss of second-order, vestibular reflex projection neurons.

## Introduction

The vestibular system processes signals generated by vestibular hair-cell mechanoreceptors of the inner ear in response to linear and angular acceleration to maintain posture, balance, and spatial orientation. The mature inner ear is a complex labyrinth including many elaborate sensory organs in a fixed configuration. However, inner ear malformation is not rare during development, despite the evolutionary significance of the labyrinths for survival. Over 40 genes affect the inner ear with many expressed during key developmental stages (1). A major hallmark of many CVDs is the formation of a malformed inner ear into a sac-like structure with the semicircular canals missing or truncated and the vestibular sensory organs relatively intact (Table 1). A sac-like inner ear is observed in CHARGE syndrome (9, 11, 23), an acronym for a set of congenital malformations: **C**oloboma (eye defects), **H**eart defects, choanal **A**tresia (nasal passage defects), **R**etarded growth, **G**enital hypoplasia, and **E**ar abnormalities. In addition, Waardenburg (15), Noonan (16), Wildervanck (17), Goldenhar (18), and branchio-oto-renal syndromes (BOR) (20) form a sac-like inner ear during development. Epidemiological studies reveal that vestibular disorders affect about 3.3 million children in the US alone (5.3–10%) (24, 25). Due to the major role that the vestibular system plays in daily living, vestibular symptoms can impose life-altering disabilities that become a major healthcare burden. CVD children may experience disorientation, confusion, and fatigue while performing daily tasks, and severe challenges in eye-hand coordination, eye tracking, and reading that may lead to difficulties in physical and intellectual development (26). Physical therapy is helpful to manage certain symptoms, but does not counter poor visual acuity or balance deficits experienced by patients with bilateral vestibular abnormalities (27, 28). Although clinical testing of CVD patients detects gross vestibular behavioral defects, testing is not sufficiently sensitive to identify the cellular sites of the pathology, within the cristae, maculae, or specific neuron subsets of the central vestibular network. To this end, animal models offer the opportunity to evaluate the origins of CVD dysfunction at the cellular level. The cellular level of analysis may offer new insight into the type and amount of vestibular adaptation necessary to restore function in CVD patients.

**Table 1:** Sac-like phenotype in human congenital vestibular disorders and animal models.

**Syndrome**	**Approach**	**Inner ear shape**	**Canals**	**Cristae** **Ampullae**	**Maculae**	**Calyces**	**VGs**	**Cochlea**	**Reference**
CHARGE	Human; *n* = 37; CT, MRI	Sac	Absent; superiorNM may be truncated	ND	ND	ND	ND	ND	(2)
CHARGE	Human; *n* = 1; CT, histopathology	Sac	Absent/ truncated	Ampullae absent	Atrophy, utricle, saccule	ND	N ND	Aud ganglion reduced to 35%	(3)
CHARGE	Human; *n* = 107; CT	Sac	Absent	ND	ND	ND	ND	ND	(4)
CHARGE	Human; *n* = 42	Sac	Absent	ND	ND	ND	ND	Short	(5)
CHARGE	Human; *n* = 18; CT	Sac	Absent/truncated	ND	ND	ND	ND	Short	(6)
CHARGE	Human; *n* = 4; CT	Sac	Absent	ND	ND	ND	ND	ND	(7)
CHARGE	Human; *n* = 13; CT	Sac	Absent	ND	ND	ND	ND	Short	(8)
CHARGE	Human; *n* = 1; histopathology	Sac	Absent	Normal; no ampullae	Utricle small; saccule normal	ND	ND	Short; no cochlea nerve or ganglion	(9) case 1
CHARGE	Human; *n* = 1; CT	Sac	Absent	ND	ND	ND		Short	(10)
CHARGE	Human; *n* = 12; CT	Sac	Absent	ND	ND	ND	ND	ND	(11)
CHARGE	Human; *n* = 7; CT; EVAR; OVAR	Sac	Absent	ND	ND	ND	ND	Short	(12)
CHARGE	Human, *n* = 1; histopathology	Sac	Absent	No cristae No ampullae	Utricle absent saccule normal	ND	Few VG	Short	(13)
CHARGE	Human; *n* = 1; histopathology	Sac	Absent	ND; no ampullae	utricle small saccule reduced	ND	Few VGs	Short	(14) Case 4
Waardenburg	Human; *n* = 151; CT; MRI	Sac	Absent or truncated	ND	ND	ND	ND	Length normal; width small and flat	(15)
Noonan	Human; *n* = 1; CT	Sac	Absent	ND	ND	ND	ND	Short	(16)
Wildervanck	Human; *n* = 1; MRI	Sac	Absent, truncated post canal	ND	ND	ND	ND	Short	(17)
Goldenhar	Human; *n* = 5; CT	Sac	Absent/truncated	ND	ND	ND	ND	ND	(18)
Goldenhar	Human; *n* = 1; CT and MRI	Sac	Absent	ND	ND	ND	ND	Short	(19)
Branchio-oto- renal	Human; *n* = 32; MRI	Sac	Absent	ND	ND	ND	ND	Short	(20)
*Chd7* mutant	Mouse; fast green: *n* = 16; neuro-filament and f-actin; *n* = 31	Variable	Highly variable/present/missing/ truncated	Superior and lateral normal; posterior patch-like	normal	present	ND	ND	(21)
ARO/s	Chicken; paint-fill, *n* = 25; Nissl sections, *n* = 8	Sac	Missing/truncated	Sup crista short A/P	utricle short A/P; saccule normal	present	VGs reduced to 62%	Often missing	(22)

*A/P, anterior/posterior extent; CT, computed tomography; MRI, magnetic resonance imaging; ND, not done; VG, vestibular ganglion cells. The table was ordered by first listing human CVDs: CHARGE, Waardenburg, Noonan, Wildervanck, Goldenhar, Branchio-oto-renal followed by animal models: mouse, chick. The most common phenotype found is listed for each reference*.

This review is focused on two main CVD phenotypes that result from gene mutation. The most common phenotype is the sac-like inner ear with the vestibular sensory organs relatively intact, whereas the less frequent phenotype forms more discrete abnormalities, such as abnormal hair-cell stereocilia. Why are there two main phenotypes? Since genes are known to be active at specific stages of inner ear development, the time of gene action determines the type of malformation. By reference to the schedule for inner ear morphogenesis in a species, the time of gene action can be predicted [chick (29–31)], [mouse (32)], and [human (33–35)]. For example, the chick otocyst forms a sac-like inner ear at embryonic day 4 (E4) (29). By E6, the superior and posterior canals emerge, with lateral canal outgrowth 6 h later. The vestibular sensory organs form *before* canal outgrowth, with the superior and posterior cristae developing at E3–3.5, macula sacculi and lateral cristae at E3.5–4, basilar papilla at E4, and macula utriculi at E4.5 (30). Thus, a sac-like, inner ear pathology results from gene mutation pin-pointed to E4–4.5 to produce a sac-like inner ear with missing or truncated canals and macula utriculi and cochlea development primarily affected. Regarding CVDs that form defects in the stereocilia linkage, the mutation must occur *after* vestibular hair cell formation that takes place in the latter half of chick embryonic development: E10 for type II hair cells and E15 for type I hair cells (36). Altogether, it is intriguing that most CVDs result from mutation of genes acting relatively early in gestation.

## Vestibular System Pathology in CVD Patients and Clinical Findings

Histological preparations of postmortem specimens have provided the most comprehensive descriptions of human inner-ear pathology in CVDs [e.g., (9)]. When inner ear structures are imaged in the clinic, the configuration of the bony labyrinths are demonstrated by computed tomography (CT), while gross details of the membranous labyrinths, fluid-filled inner-ear spaces, and vestibular nerves are seen using magnetic resonance imaging (MRI). Dysfunction in the vestibular system of CVD children can be investigated in the clinic by testing directly the vestibular reflex pathways. For example, ocular and cervical vestibular evoked myogenic potentials reveal utricular or saccular dysfunction, while bithermic caloric tests, earth vertical axis rotation, and head impulse tests demonstrate semicircular canal pathology (27).

### CVDs Forming a Sac-Like Inner Ear

#### Charge Syndrome

The vast majority of CVD cases in the literature are based on evaluations of CHARGE syndrome patients, with most studies containing one or a few patients. Like most CVDs, CHARGE syndrome has a low incidence (1/10,000 births) [e.g., (37)]. CHARGE syndrome patients develop a sac-like inner ear with the semicircular canals missing or truncated, and few defects in the cristae or maculae (9). Vestibular sensory organs contacted by the anterior vestibular nerve tend to show more abnormalities, with the utricular maculae often decreased in overall extent (9). Also, the cochlea may be shortened or absent. In some CHARGE syndrome patients, the auditory or vestibular ganglion is small or absent (3, 13, 14). From vestibuloocular reflex (VOR) testing, combined with CT and MRI imaging, CHARGE syndrome patients present relatively normal otolith function, but lack canal activity (11, 12, 38). Clinically, CHARGE syndrome patients present with mild to profound hearing loss and balance deficits (39).

#### Waardenburg Syndrome

With the incidence at 1/42,000, this syndrome is one of the most common causes of congenital, syndromic deafness (40). Although clinically and genetically heterogeneous, the disorder is characterized by sensorineural hearing loss and pigmentation deficits (15): Depending on symptoms, four subtypes are identified, with vestibular deficits present in WS2 and WS4, and SOX10 mutation linked to these two subtypes (41). Temporal bone scans reveal deformed, highly variable semicircular canals, enlarged vestibule, and small, flattened cochlea (15). All three semicircular canals are truncated or absent. In one study, three patients lacked all three semicircular canals, while two other patients showed only unilateral canal loss (15). In another study, the semicircular canals were absent bilaterally (42). While vestibular dysfunction in Waardenburg syndrome is not well-studied in the clinic, one study did show that patients missing all three semicircular canals presented with complete loss of vestibular function, while other patients with one hypoplastic canal showed some vestibular function, suggesting that truncated canals and a sac-like inner ear are capable of generating and transmitting vestibular signals (15). In a case study of 22 Waardenburg patients, those patients with symptoms of vertigo, dizziness or imbalance, showed abnormal VOR and vestibulospinal tests (43).

#### Noonan Syndrome

Noonan syndrome (NS) is a relatively common genetic disorder (incidence: 1/1,000 to 1/2,500) (44), producing multiple abnormalities, including short stature, broad neck, and developmental delays. *NS* results from an autosomal dominant gene mutation that occurs in multiple loci. In a report from a single NS patient, CT imaging revealed absence of the semicircular canals and VOR testing showed no measurable vestibular function (16).

#### Wildervanck Syndrome

Wildervanck syndrome, also called cervico-oculo-acoustic syndrome, is a rare congenital disorder (incidence, 1/1,000,000; Orphanet), characterized by fusion of the cervical vertebrae, oculomotor dysfunction causing horizontal gaze paralysis, and congenital deafness. Wildervanck syndrome almost exclusively affects females, since the hemizygous male is lethal (45). In a study of one Wildervanck syndrome patient, both inner ears lacked all three semicircular canals, with the vestibule missing on one side (17). Recurrent attacks of dizziness are experienced in some Wildervanck patients.

#### Goldenhar Syndrome

Goldenhar syndrome, also known as oculo-auriculo-vertebral syndrome, is a congenital disorder (incidence, 1/3,500 to 1/5,600) (46) in which the patients present with diverse malformations, with the most characteristic features ocular anomalies, such as microphthalmia, external, middle, and inner ear malformation, and vertebral defects. Less attention has been paid to the inner ear malformations in this syndrome. One study of 21 patients showed that 33% of patients presented with inner ear malformations, including cochlear hypoplasia, underdeveloped or missing semicircular canals, enlarged vestibular aqueduct, and enlarged internal auditory meatus (46). Other studies report a variable number of semicircular canals in one or both ears (18, 19). Since conductive and sensorineural hearing deficits are prominent in Goldenhar patients, clinics have focused on managing their hearing loss rather than addressing the vestibular defects.

#### Branchio-Oto-Renal Syndrome

BOR syndrome (incidence, 1/40,000; Orphanet) is an autosomal dominant congenital disorder, resulting from mutation of *SIX1* or *EYA1* genes that affect development of the ear, kidney, and neck [for review, see (47)]. There is considerable variability in the presence, type, and severity of the clinical abnormalities, but hearing loss is the most prevalent symptom (48). CT and MRI of the temporal bone reveal inconsistent inner ear abnormalities, but cochlea hypoplasia or dysplasia, vestibular aqueduct enlargement, and absence or hypoplasia of the semicircular canals are reported most often (49, 50). Evidence of cochleovestibular nerve malformation is also found (51), but no descriptions are provided on the condition of the vestibular sensory organs or vestibular symptoms in patients.

### CVDs Forming Diverse Vestibular Inner Ear Defects

#### Enlarged Vestibular Aqueduct Syndrome

Enlarged vestibular aqueduct syndrome (EVA) is a common congenital inner ear disorder (incidence, 1/100–1,300) (52, 53). Diagnosis is made primarily from CT and MRI scans that show an enlarged vestibular aqueduct or enlarged endolymphatic duct and sac, with no abnormalities apparent in the semicircular canals or vestibular sensory organs (54). EVA primarily produces mild to profound sensorineural hearing loss accompanied by mild imbalance or episodic vertigo (54). In a study of 106 patients, 45% presented with vestibular symptoms (55).

#### Otogelin Disorder

Otogelin is an extracellular, N-glycosylated protein composing the fibrillar network linking the tectorial membrane to the outer hair cells and anchoring otoconia and cupula to the underlying neuroepithelium (56). Mutation of the gene encoding otogelin causes autosomal-recessive, non-syndromic, moderate hearing loss associated with severe imbalance, delayed motor development, and dizziness (57, 58). CT scans do not reveal inner ear abnormalities.

#### Usher Syndrome

Usher syndrome is the leading cause of combined visual and hearing loss, with vestibular function abnormal in certain genotypes (59). The syndrome is clinically and genetically diverse, with a low incidence of 1.6–4.4/10,000 (60). Hearing and vestibular dysfunction result from defective tip linkages connecting the hair-cell stereocilia that prevents sensory transduction (61). There are three types of Usher syndrome. Type 1 patients have profound hearing loss and severe balance deficits resulting in delays in sitting and walking. Type 2 patients show moderate-to-severe hearing loss that may be accompanied by balance deficits. Type 3 patients show progressive hearing loss, retinal degeneration, and unpredictable vestibular dysfunction.

#### Pejvakin Disorder

*PJVK* is a protein expressed in hair and supporting cells of the inner ear and spiral ganglion neurons that has been shown to be necessary to maintain stereocilia structure in outer hair cells (62). In mice, PJVK mutation is linked to an autosomal recessive, non-syndromic hearing loss and balance deficits (63). Variability in PJVK mutations underlies the diverse phenotypes in these patients. Clinical reports routinely describe the nature of the hearing loss, but do not report on balance defects in these patients.

## CVD Animal Models

### CVD Animal Models Forming a Sac-Like Inner Ear Phenotype

#### Charge Syndrome

*S*ince most CHARGE syndrome patients show heterozygous mutation of the chromodomain helicase DNA binding protein 7 gene *CHD7*, animals with heterozygous *Chd7* mutation are a popular research model (64). At present, there are two animal models commonly used to study CHARGE syndrome, Chd7 mutant mice and Chd7 mutant zebrafish. The *Chd7* gene influences many downstream genes involved in inner ear development (65) that may explain the diversity of inner ear phenotypes. At present, the downstream genetics of CHARGE syndrome are not sufficiently known to generate a mutant animal model with a consistent inner ear phenotype.

*Chd7 mutant mice* show diverse semicircular-canal malformations (21). The superior canal is often normal, while the lateral canal is absent or has a reduced diameter in diverse configurations (21). The posterior canal often has a reduced diameter. The lateral ampullae may have a small width. The cristae and maculae may develop abnormal shapes. Beside the normal saddle-shape, the lateral cristae may have a smaller width and the posterior cristae may appear with round-patch-like or flattened epithelium that lacks calycine endings and vestibular afferents. Like CHARGE syndrome patients, vestibular ganglia are small in *Chd7* mutant mice (66). Finally, *Chd7* mutant mice may form a small ocular lens and display cerebellar hypoplasia (67, 68). Since the visual system and cerebellum interact intimately with the vestibular system, their pathology makes it difficult if not impossible to sort out the origin of vestibular deficits in *Chd7* mutant mice. *Chd7* mutant mice perform rapid, bidirectional circling movements, indicating bilateral defects in the lateral canal (21).

*Chd7 mutant zebrafish* primarily show defects in the otoliths, forming either asymmetric otoliths or one irregular otolith (69). The general size and shape of the semicircular canals are also abnormal. Thus, Chd7 mutation in zebrafish does not mirror the inner ear phenotype found in CHD7 patients.

#### Waardenburg Syndrome

Various gene mutations can produce WS, including mutation of PAX3, MITF, EDNRB, EDN3, and SOX10 genes [for review, see (15, 70)]. For example, the panda pig is used to study MITF-M expression, which is mutated in WS type 2. Unlike the human disorder, this model loses vestibular hair cells in the saccule, without defects in the utricle or cristae (70).

#### Noonan Syndrome

NS belongs to a group of clinically-related developmental disorders that result from mutations in the RAS signaling pathway [for review, see (71)]. While there are studies using RAS mutant mice to study NS, vestibular inner ear abnormalities have not been evaluated [e.g., (71, 72)].

#### Wildervanck Syndrome

No studies have been reported on an animal model to study this disorder.

#### Goldenhar Syndrome

The gene ZYG11B, thought to be defective in Goldenhar syndrome, is under study in zebrafish (73).

#### BOR Syndrome

*Six1* heterozygous mutant mice have been used to study inner ear malformation in BOR syndrome (74). Unlike the human disorder, the mutant mouse model lacks all the sensory organs in the inner ear, along with a malformed saccule, no posterior ampulla, and truncated or missing posterior semicircular canal.

#### A Universal Model for the Sac-Like Inner Ear Phenotype

**Figure 1:**
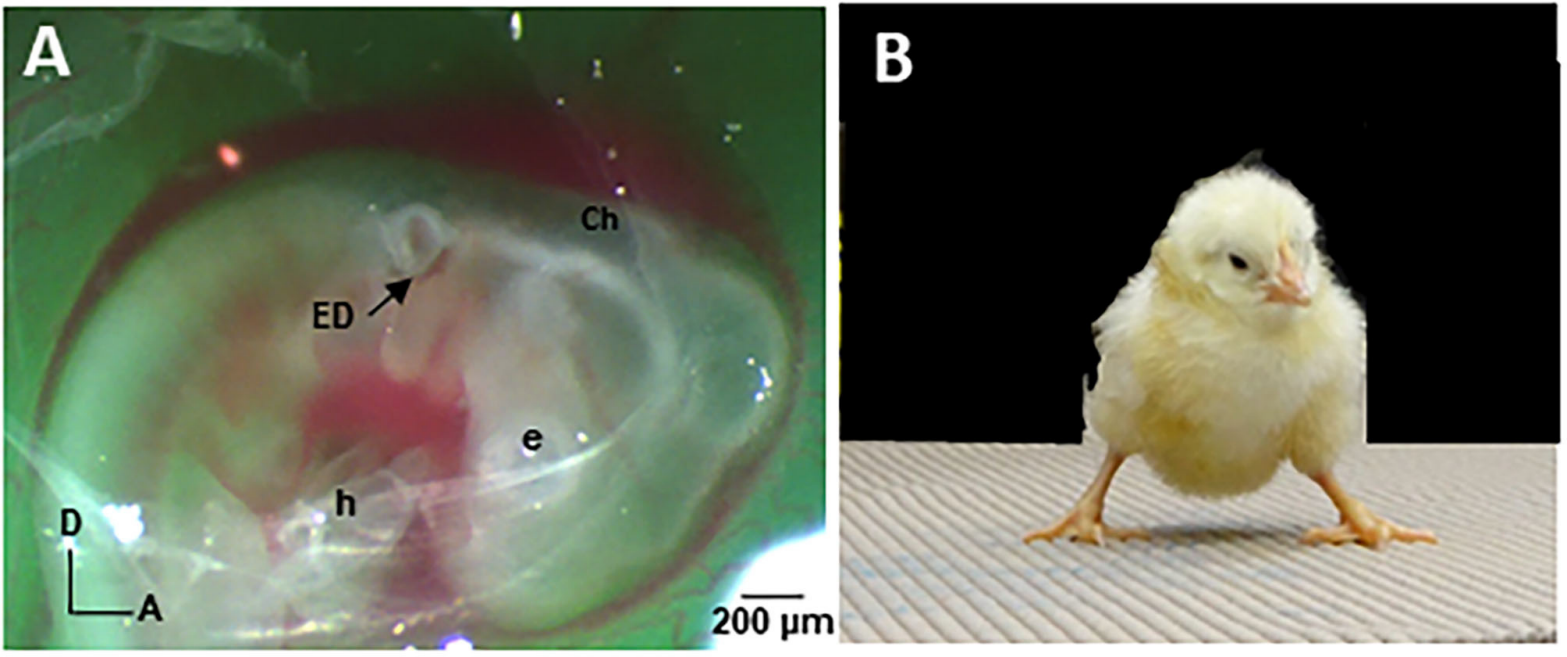
**(A)** E2 chick in ovo viewed through a shell window under a stereo dissecting microscope (Zeiss Discovery.V8) after otocyst rotation. Fast green dye was injected to improve otocyst visibility. After tearing open the chorion (Ch) and amnion with forceps, the otocyst was cut free from the surrounding epithelium with a tungsten needle (10 μm tip diameter; curved tip) and glided posteriorly along the epithelial surface before rotating it 180° in the anterior-posterior and dorsal-ventral axes. The rotated otocyst was returned to the epithelial slot. Note that the endolymphatic duct (ED) normally situated on the dorsal surface of the otocyst is located on the ventral surface after rotation. The shell window was sealed with tape and the egg reincubated at high humidity (70%) without egg turning (75). E, eye; h, heart. **(B)** Five-day-old hatchling ARO/s chick. Note the widened base of the feet after performing the righting reflex, indicating stress placed on the balance system (22).

The chick has a long and distinguished history in biomedical research as a model to study inner ear and central nervous system development. To further advance understanding of the sac-like inner ear pathology in CVD patients, we designed and implemented a CVD animal model by surgically rotating the developing inner ear, or otocyst, in 2-day-old chick embryos (E2) (22, 75). The right otocyst can be readily accessed in E2 chick embryos in *ovo* for microsurgical manipulation (Figure 1A). **A**nterior-posterior axis **R**otation of the **O**tocyst 180° produces a **s**ac-like inner ear on the right side, called the “ARO/s chick.” This model is reproducible in 85% of cases, forming a sac-like inner ear that mirrors the common inner ear pathology found in many CVD patients. Thus, the ARO/s chick develops without introducing genetic mutations that affect other targets beside the inner ear. Altogether, the ARO/s chick offers a highly tractable model to study the deterministic connections between the malformed inner ear and vestibular neural circuitry abnormalities/plasticity. Like CVD children, ARO/s hatchlings experience balance and walking problems (Figure 1B).

By E13, the chick semicircular canals, vestibular sensory organs, and certain vestibular nuclei neurons have differentiated so that they provide identifiable experimental targets (29, 76, 77). The membranous labyrinths can be imaged using opaque paint to view canal size and cochlea configuration compared to the normal counterparts (75). ARO/s chicks form a sac-like inner ear with the semicircular canals missing or truncated (22). Nissl-stained, serial tissue sections show that the vestibular sensory organs associated with the anterior vestibular nerve are more often affected in ARO/s chicks (22), like CVD patients (9). In preliminary studies of the vestibular ganglion (VG), VG cell number is reduced to 62% on the rotated side compared to the non-rotated side, with vestibular ganglion cell number on the non-rotated side similar to controls (78). The advantages of the ARO/s chick model include a reliable source of embryos with well-characterized phenotype, utility of the non-rotated inner ear as an internal control, and lack of pathology in related neural systems that could convolute the analysis and interpretation of the effects of the sac-like inner ear pathology on vestibular neural circuitry development. In addition, the tangential vestibular nucleus (TN) is a major vestibular nucleus whose principal cells are vestibular reflex neurons. In E13 ARO/s chicks, the number of principal cells on the rotated side was reduced to 35% of the normal number in control chicks (22). This indicates that the sac-like inner ear pathology produces major developmental abnormalities in the downstream vestibular reflex pathways. Finally, the vestibular behaviors observed in 5-day-old hatchling (H5) ARO/s chicks are consistent with a unilateral vestibular deficit (79). ARO/s chicks with a right, sac-like inner ear tilt their head to the right, but do not display eye deviations or nystagmus at rest. However, after performing the righting reflex, ARO/s hatchlings stumble and close their eyes, in contrast to the normal hatchlings. After performing the righting reflex, ARO/s hatchlings stand with a widened base, suggesting balance deficits (Figure 1B) (22).

### CVD Animal Models Forming Diverse Vestibular Inner Ear Abnormalities

#### EVA Syndrome

Mutation of *SLC26A4* gene in mice results in profound hearing loss, vestibular abnormalities, and enlarged endolymphatic spaces throughout the inner ear (80). In the utricle and saccule, the maculae are degenerated with the near complete absence of the otoconia. Unlike the human disorder, macular hair cells in the mouse model continue to degenerate as postnatal development progresses.

#### Otogelin

*Rock solo*^*AN*66^ is a zebrafish mutant of the glycoprotein otogelin (81). It is interesting that balance and postural deficits present in young mutant larvae later disappear with no permanent vestibular deficits, unlike the human disorder.

#### Usher Syndrome

*Ush 1c* mutant mice are defective for harmonin-b, a protein located in the tip links of hair-cell stereocilia (61). Vestibular sensory evoked potentials are absent, indicating malfunction primarily in the utricular macula (82). In another Usher 1 mutant mouse model, SANS, the defect occurs in a protein involved in stereocilia differentiation and VOR testing reveals that canal and otolith activity are absent (83).

#### Pejvakin Syndrome

Several *Pjvk-mutant* mice models have been produced that show phenotypic variability (62). In studies of Pjvk-flox mutation, pejvakin was found to be localized to the base of the stereocilia rootlets in cochlear outer hair cells (63). Mice develop normal rootlets, but the stereociliary bundles and mechanotransduction are defective, resulting in deafness. In a study using *Pjvk G292R* mutant mice, progressive hearing loss occurs, with vestibular dysfunction indicated by head tilt, circling behavior, and abnormal swimming and balance (62). Fluorescence microscopy shows that the vestibular hair cells are intact, but the vestibular ganglion neurons are lost progressively.

## Discussion

### Benefits and Limitations of CVD Animal Models

Animal models offer living, non-human subjects to understand human disease without compromising human life. Often, animal models are genetically engineered. Despite ortholog gene mutations, most animal models do not accurately replicate the human pathology because disease phenotypes result from complex interactions between genes and environment. Indeed, a phenotype does not result from disrupting one gene, but a cascade of genes whose dosage of alleles may vary [for review (84)]. Nonetheless, many advantageous drugs, treatments, and cures for human diseases have resulted from using animal models.

Animal models are most advantageous when they share the same cause and symptoms for a disease as the patients with the disease. Alternately, there are animal models that share the same symptoms, but not the same etiology, for example, the ARO/s chick. ARO/s chicks are formed by microsurgical manipulation of the otocyst rather than genetic mutation, but subsequent development of the animal model produces a similar sac-like inner ear phenotype with minimally-disrupted, vestibular sensory organs that parallels the human phenotype found in many CVDs. Finally, human genetic disorders show considerable variability in the presence, type, and severity of the abnormalities, even among family members with the same mutation, so that a specific mutation is not associated with a similar clinical presentation [e.g., (85)]. This holds true for animal models, as seen in the diversity of inner ear phenotypes found in *Chd7* mutant mice used to study CHARGE syndrome.

### Is There an Optimal Window for Therapeutic Intervention?

Genetic testing for potential inherited mutations can be performed early in fetal development so that therapeutic intervention can be initiated prenatally, possibly before vestibular dysfunction emerges (86). The time for treatment depends on the time when the pathology appears, which can be assayed in an animal model and then extrapolated to the equivalent human gestational age. Treatment delivered locally in the postnatal inner ear avoids potential toxic effects of systemic delivery [e.g., (83)]. In addition, our chick model offers easy access to the *embryonic* inner ear *in ovo* without compromising continued development. The fluid-filled, developing inner ear also provides a suitable vehicle to inject drugs prenatally to determine whether balance can be rescued. After hatching, balance can be tested in the ARO/s hatchling (22).

### What Is the Underlying Pathophysiology of CVDs?

Why is canal function lost on clinical VOR testing of CVD children? Canal sensation relies on endolymph fluid movement in the canal to convert angular head movements into displacement of the stereocilia on the apical surface of the vestibular hair cells. Endolymph displacement on head movements depends on key geometric features of the canals to maintain mechanical sensitivity, including canal radius and canal length (87). During Xenopus development, rotatory head movements generate signals in the crista only after the semicircular canals have acquired a minimal diameter, despite the presence of functional activity in other components of the vestibular reflex circuitry, including the hair cells, central vestibular pathways, and extraocular eye muscles (88). Blocking individual semicircular canals, or “canal plugging,” is an approach that was used to determine the role of individual canals (89). At low rotational frequencies (<2 Hz), endolymph movement is reduced greatly in a plugged canal, with only minimal hair-cell stimulation in the blocked canal, but normal function preserved in the other canals and otoliths (90, 91). At present, canal plugging is a surgical approach performed on patients to alleviate vestibular symptoms resulting from canal dehiscence. To what extent does the sac-like inner ear, with or without truncated canals, affect vestibular signaling? Does synaptic plasticity in the peripheral or central vestibular neural circuitry impact vestibular signaling in CVD patients? These are some questions for animal models to address that may translate into clinical practice.

There have been no systematic studies of connectivity in the peripheral or central vestibular neural circuitry of CVD animal models. The ARO/s chick is now available to interrogate the anatomical and functional modifications, since the model is reproducible and recapitulates salient features found in CVD patients (22, 75). Developmental studies have been performed on normal and vestibular ganglionectomized chicks at critical pre- and post-natal ages (76, 77, 79, 92–95). TN principal cells offer a structurally-uniform subset of vestibular reflex projection neurons to determine whether the orderly ingrowth of the canal and otolith fibers occurs and whether passive and active membrane properties emerge within the normal time frame during ARO/s chick development. Is normal synaptic transmission conserved in principal cells of ARO/s chicks, or do major changes occur in the spontaneous excitatory and inhibitory synaptic events (93–95)? VOR testing using Earth vertical axis rotation for canal function and static tilting platform for otolith function has been performed on animal models and offers a powerful research tool to evaluate potential therapeutic intervention (96, 97). Altogether, the outcome of animal model experiments could provide novel insights into the consequences of CVDs on the development, maintenance, and plasticity of the vestibular neural circuitry that will modify our thinking on how to treat the disorders. This includes insights into whether the neural circuitry may benefit from vestibular implant technology or specific pharmacological treatments (28, 98–100).

## Author Contributions

KP is responsible for the conception of the review. KP, NB, JH, MB, and AP are responsible for the design of the review and contributed to manuscript revision, read, and approved the submitted version. KP and NB are responsible for writing the first draft of the review. All authors contributed to the article and approved the submitted version.

## Funding

This work was supported in part by NIH grant R01 DC019369 (KP), The George Washington University School of Medicine and Health Sciences Research Funds (KP), Robert Vincent Research Fellowship of the Columbian College of Arts and Sciences of The George Washington University (NB), Centre National de la Recherche Scientifique (MB), Université de Paris (MB), IdEx Université de Paris ANR-18-IDEX-0001 (MB), and Intellectual and Developmental Disabilities Research Center [NICHD Grant U54-HD090257 (AP)].

## Conflict of Interest

The authors declare that the research was conducted in the absence of any commercial or financial relationships that could be construed as a potential conflict of interest.

## Publisher's Note

All claims expressed in this article are solely those of the authors and do not necessarily represent those of their affiliated organizations, or those of the publisher, the editors and the reviewers. Any product that may be evaluated in this article, or claim that may be made by its manufacturer, is not guaranteed or endorsed by the publisher.
